# DNAJB9 Fibrillary Glomerulonephritis With Membranous-Like Pattern: A Case-Based Literature Review

**DOI:** 10.7759/cureus.47862

**Published:** 2023-10-28

**Authors:** Nikolaos Sabanis, Paraskevi Liaveri, Virginia Geladari, George Liapis, George Moustakas

**Affiliations:** 1 Department of Nephrology, General Hospital of Trikala, Trikala, GRC; 2 Department of Nephrology, General Hospital of Athens “Georgios Gennimatas”, Athens, GRC; 3 Department of Internal Medicine, General Hospital of Trikala, Trikala, GRC; 4 Department of Pathology, National and Kapodistrian University of Athens, Athens, GRC

**Keywords:** electron microscopy, nephrotic range proteinuria, membranous-like pattern, dnajb9, fibrillary glomerulonephritis

## Abstract

Fibrillary glomerulonephritis (FGN) is a rare immune-mediated glomerular disease traditionally characterized by the presence of amyloid-like, randomly aligned, fibrillary deposits in the capillary wall, measuring approximately 20 nm in diameter and composed of polyclonal IgG. FGN is usually a primary disease with no pathognomonic clinical or laboratory findings. More than that, on light microscopic evaluation, it can receive various histological patterns, rendering its diagnosis indistinguishable. However, the identification by immunohistochemistry of a novel biomarker, DNA-J heat-shock protein family member B9 (DNAJB9), has created a new era in FGN diagnosis even in the absence of electron microscopy.

Typically, most patients manifest various degrees of renal insufficiency, hypertension, microscopic hematuria, proteinuria, and occasionally frank nephrotic syndrome. The prognosis is usually severe and progression to end-stage kidney disease (ESKD) is the rule, given that no specific treatment is available until now, despite the fact that in small studies rituximab-based therapy seems to alleviate the severity and improve the disease progression.

Herein, we report the case of a 63-year-old Caucasian man presenting with uncontrolled hypertension, headache, shortness of breath, and lower limb edema. Diagnostic evaluation revealed mild deterioration of kidney function, nephrotic range proteinuria, and faint IgGκ monoclonal bands in serum and urine immunofixation. After negative meticulous investigation for secondary nephrotic syndrome causes, the patient underwent a kidney biopsy. Biopsy sample showed two glomeruli with mesangial expansion and thickened glomerular basement membrane (GBM) on light microscopy, a pattern masquerading as membranous nephropathy stage III-IV, while IgG and C3 were 1-2+ on GBM and mesangium in immunofluorescence. Thickened GBM with fibrils on electron microscopy were found, while DNAJB9 in immunohistochemistry was positive, confirming FGN.

Once diagnosis of FGN was made, a combination of steroids with rituximab was initiated while the patient was receiving the standard anti-hypertensive therapy, simultaneously with a sodium-glucose cotransporter-2 (SGLT2) inhibitor. The 12-month follow-up showed approximately 85% decrease in proteinuria alongside stabilization of kidney function and blood pressure normalization.

Hence, in this article, we aim to highlight that DNAJB9-associated FGN may mimic membranous glomerulopathy stage III-IV on light microscopy, especially when a small kidney sample with extensive involvement by fibrils of GBM is examined. Moreover, we underscore the fact that ultramicroscopic examination is of crucial importance in the differential diagnosis of glomerular deposition diseases and that DNAJB9 identification on immunohistochemistry consists of a revolutionary and robust biomarker in FGN diagnosis.

## Introduction

Fibrillary glomerulonephritis (FGN), first described in 1977 by Rosenmann and Eliakim while investigating the case of an Arab patient presenting with nephrotic syndrome, represents a quite rare glomerulopathy associated with immune complexes [1,2]. Diagnosis of FGN typically ranges from 0.6% to 1% of native kidney biopsy specimens and is characterized by the glomerular deposition of randomly oriented fibrils, measuring 10-30 nm in thickness, visible on electron microscopy [2,3].

Most cases occur in Caucasian patients in the sixth decade of their life, with gender predominance in females (male-to-female ratio 1:1.8). However, specific reports reveal male predominance in some countries such as Greece [3-5].

Patients usually present with proteinuria, occasionally frank nephrotic syndrome, microscopic hematuria, hypertension, and kidney function impairment [3,4]. FGN can present either as a primary glomerulopathy or a secondary manifestation of a systematic disease. Specifically, it has been reported that FGN has a strong correlation with diabetes mellitus, autoimmune diseases, hepatitis C and several virus infections, and dysproteinemia, such as multiple myeloma and hematologic malignancies [3]. Prognosis is poor, with approximately one-half of patients progressing to end-stage kidney disease (ESKD) in a mean time of four years [6].

In our case, we report a 63-year-old male who presented at the Emergency Department complaining of uncontrolled hypertension and bilateral edema of the lower extremities. The initial laboratory exams revealed nephrotic range proteinuria and mild renal insufficiency. The patient underwent a complete investigation for secondary nephrotic syndrome causes, and eventually a kidney biopsy was performed. The biopsy results unexpectedly confirmed the diagnosis of FGN on electron microscopy and immunohistochemistry, whereas on light microscopy, the small kidney sample included five glomeruli in total, presenting as membranous-like nephropathy stage III-IV.

Once diagnosis was made and despite the doubtable outcomes of current immunosuppressive therapies, a combination of steroids with rituximab was initiated alongside the administration of an SGLT2 inhibitor. After 12-month surveillance, the follow-up revealed an almost 85% decrease in proteinuria and stabilization of kidney function.

Hence, in this article, we report a case of DNAJB9-associated FGN masquerading as membranous glomerulopathy on light microscopy. In this context, we discuss diagnostic challenges and pitfalls and highlight the fact that a membranous-like morphologic variant may be seen in cases of extensive glomerular depositions of fibrils, especially when microscopy is performed in a small kidney sample. Finally, in the light of identifying a novel biomarker by immunohistochemistry, DNAJB9, we aim to synthesize current knowledge concerning FGN pathogenesis, diagnosis, and management.

## Case presentation

A 63-year-old Caucasian male presented to the Emergency Department complaining about high blood pressure, headache, shortness of breath, and bilateral pitting edema of the lower extremities. He reported poor compliance to antihypertensive treatment in the last few months and no history of illicit drug intake, vaccination, or infection. Furthermore, he reported a negative family history of kidney disease and malignancy. His previous medical history included hypertension during the last 20 years, former morbid obesity, smoking (50 pack-years), and a 10-year history of undetermined mild proteinuria (1.2 g/24h), which was arbitrarily attributed to his longstanding hypertension; the diagnosis was made without a kidney biopsy. The patient’s daily medication included irbesartan 300 mg/hydrochlorothiazide 12.5 mg once daily (o.d.), manidipine 20 mg o.d., moxonidine 0.4 mg o.d., nebivolol 5 mg o.d, bromazepam 3 mg o.d, and sertraline 50 mg o.d.

On admission, the patient was afebrile, his heart rate was 85 beats per minute, his oxygen saturation was 99% on ambient air, and severe blood pressure elevation was measured (BP=235/140 mmHg) on both upper limbs.

Afterward, a meticulous clinical and laboratory evaluation, including electrocardiography, transthoracic echocardiography, troponin levels, and CT thoracic angiography, was performed, excluding the diagnosis of acute hypertension-mediated organ damage. Similarly, brain CT imaging excluded acute events such as stroke, cerebral hemorrhage, and hypertensive encephalopathy. Fundus examination revealed stage IΙ chronic hypertensive retinopathy, while no findings compatible with a hypertensive emergency, such as retinal bleeding or papilledema, were present. Routine laboratory blood tests showed mild kidney function impairment with serum creatinine and urea levels at 1.25 mg/dl and 55 mg/dl, respectively, as well as hypokalemia with potassium levels at 3.26 mEq/L. Schistocytes were not detected during the examination of the peripheral smear, as well as other laboratory findings compatible with microangiopathic hemolytic anemia. Urinalysis showed yellow, clear urine with the presence of 3+ protein and 2+ hemoglobin. Examination of urine sediment revealed microscopic hematuria (5-7 erythrocytes) without red blood cell casts and 2-4 leukocytes per high-power field. In addition, hyaline-granular casts were observed in urine microscopy.

Renal ultrasonography showed bilaterally equal kidney size and normal renal parenchymal echogenicity without evidence of hydronephrosis or obstructive uropathy. A complete ultrasound Doppler evaluation of the entire renal vasculature was performed during the next days, showing significantly increased renal resistive index bilaterally (RRI=1.54 in the right and RRI=1.17 in the left kidney), values indicative of diffuse microvascular atherosclerotic abnormalities while direct and indirect criteria for renal artery stenosis were negative.

Thereafter, the patient was admitted to the Internal Medicine Department for further investigation with modified antihypertensive therapy. During hospitalization, his medication consisted of nifedipine 20 mg every eight hours, moxonidine 0.3 mg twice daily (b.i.d), terazosin 2 mg o.d., isosorbide mononitrate 20 mg b.i.d., irbesartan 150 mg b.i.d., furosemide 40 mg o.d, febuxostat 80 mg o.d., and sertraline 100 mg o.d.

On Day 2, a 24-hour urine collection showed heavy proteinuria with protein excretion at 11.67 g/24h. Laboratory exams the same day revealed worsening of kidney function with serum creatinine 1.37 mg/dl and urea 67 mg/dl, total serum protein 6.48 mg/dl, and albumin 3.36 mg/dl, indicative as mild hypoalbuminemia, as well as normal coagulation pathway and dyslipidemia. Due to the nephrotic range proteinuria, a meticulous investigation of primary and secondary nephrotic syndrome causes was arranged.

CT of the chest, abdomen, and retroperitoneal area with the use of intravenous contrast did not illustrate the presence of malignancy, adrenal gland adenoma or hyperplasia, stenosis of renal vessels, or any other pathological findings. Moreover, urine tests for the detection of vanillylmandelic acid and metanephrines were within normal values, precluding the diagnosis of pheochromocytoma. Thereafter, the patient underwent endoscopic evaluation of the upper and lower gastrointestinal tract. The presence of a submucosal rectal protrusion was the only pathologic finding; however, biopsies were negative for malignancy or amyloidosis.

Over the next days, we obtained the results of tumor biomarkers (CEA, Ca 19.9, Ca15.3, Ca125, AFP, PSA), thyroid testing (TSH, FT3, FT4, anti-TG, anti-TPO), and serum viral serologies (HBV, HCV, and HIV), and all were revealed to be negative. Furthermore, serum and urine immunology testing (IgG, IgM, IgA, ANA, p-ANCA, c-ANCA, C3, C4, anti-dsDNA, rheumatoid factor, ACA, β2GPI, immunoglobulin-free κ/λ chains, κ/λ ratio, Bence-Jones urine protein) pointed out slight positivity for ANA (1/160). The remainder of the results were negative. Serum and urine electrophoresis and immunofixation were also delivered on Day 2, with the results showing the presence of IgGκ faint monoclonal bands on both serum and urine immunofixation.

Meanwhile, the patient was in a stable condition with his blood pressure lowered to 150/95 mmHg, and no further deterioration of kidney function was recorded. Then, the patient was transferred to a tertiary hospital in order to perform a CT-guided kidney biopsy.

**Figure 1:**
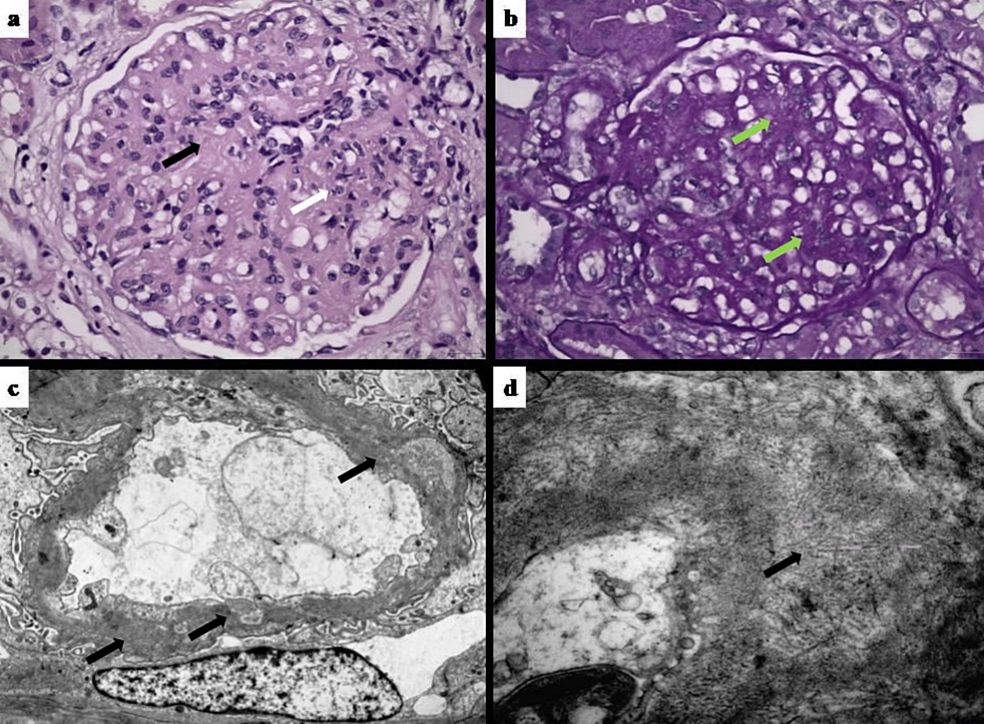
(a) Mild to moderate mesangial expansion (black arrow) with mesangial cell proliferation (white arrow) and GBM thickening (H&E X 400). (b) Mild mesangial expansion with GBM thickening (green arrows) (PAS X 400). (c) Ultrastructural appearance: intramembranous deposits in GBM, along with GBM remodeling and thickening (black arrows), reminiscent of “holes”/lucencies seen in membranous stage III-IV (uranyl acetate X 5600). (d) Intramembranous randomly arranged non-branching fibrils (black arrow) of ~16-21 nm in diameter, in higher magnification, suggestive of fibrillary GN (uranyl acetate X 22000)

We obtained two kidney biopsy cylinders, of which, the first sample, including five glomeruli, was sent for light microscopy, the second one consisting of four glomeruli was sent for electron microscopy, while a third small sample was appropriately processed for immunofluorescence (IF). Light microscopy showed three globally sclerosed glomeruli and mild glomerular size increase in two glomeruli with focal sclerosis and compaction, mild to moderate mesangial expansion accompanied by mild mesangial proliferation, and focal extensive thickness of glomerular basement membrane (GBM) with segmental vesicular degeneration (Figure 1a-1b). Additionally, mild to moderate lesions of interstitial fibrosis and tubular atrophy were observed, corresponding to 25% of renal interstitium, as well as limited foci of acute tubular injury characterized by tubular dilatation, markedly flattened epithelium, and intratubular hyaline casts. Renal vasculature changes revealed mild to moderate arteriolar hyalinosis lesions and moderate arteriosclerotic lesions with intimal fibroelastosis of larger arteries. Staining for Congo red was negative. IF revealed the presence of 1-2+ IgG, +1 IgM, 2-3+ C3, and +2 λ-chain immune depositions on the glomerular membrane and the mesangium, on a linear band-like pattern. Moreover, traces of IgA, C1q, and κ deposits were present. Electron microscopy, as a “God from the machine,” revealed extensive thickness and remodeling of GBM, characterized by the formation of a considerable amount of new basement membranes separated by lucencies. Thus, GBM remodeling with double contour appearance and lucencies formation was considered the long-term effect of immune-complexes entrenchment. More than that, compounds of randomly organized fibrils were observed mainly on glomerular membranes (measuring ~20 nm in diameter) coexisting with focal granular deposits, while limited fibrillary deposits were present in the mesangium (Figure 1-1d). Yet, the diagnosis of FGN was totally confirmed after immunohistochemical staining for DNAJB9, which revealed strongly positive alongside the GBM and segmentally on the mesangium. Meanwhile, PLA2R-antibody results were also negative.

Taking into consideration the conflicting results of immunofixation showing the presence of faint IgGκ monoclonal bands in both serum and urine and the biopsy results on IF revealing the predominance of λ chain deposits, the suspicion of an underlying dysproteinemia or hematologic malignancy should be excluded. Under this perspective, a bone marrow biopsy was arranged. Histological results did not reveal bone marrow infiltration by monoclonal lymphocytic or plasmacytic population, while karyotype analysis was normal. Fluorescence in situ hybridization (FISH) testing for the detection of TP53 gene deletion, del (17p13.1), and FGFR3 gene rearrangement was also negative, excluding the diagnosis of multiple myeloma.

**Figure 2:**
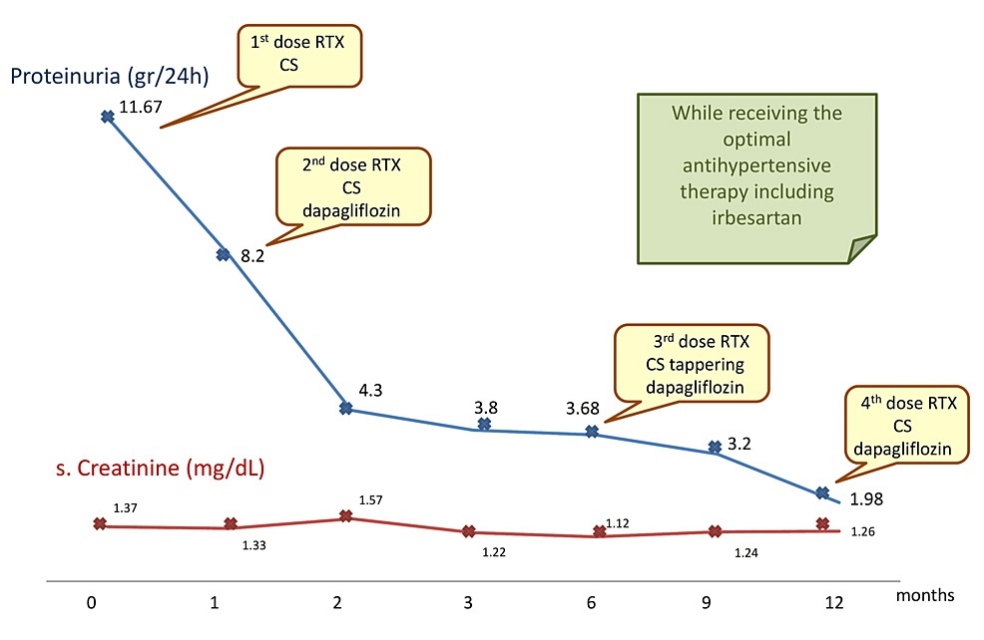
Serum creatinine and 24-hour proteinuria changes over a 12-month follow-up period RTX: rituximab, CS: corticosteroids

Throughout the meticulous investigation, none of the secondary causes of FGN (diabetes mellitus, autoimmune diseases, hepatitis C infection, dysproteinemia, and malignancies) were detected in our patient and, therefore, primary FGN was confirmed. Till now, there are limited data about the efficacy of the available therapeutic options in FGN. However, we decided to initiate our patient on a combination of steroid treatment with rituximab, while an SGLT-2 inhibitor (dapagliflozin 10 mg) was administered as an additional nephroprotective treatment from the second month. An initial oral dosage of 32 mg methylprednisolone for two months, which was further tapered over the next 10 months, was given to the patient. Simultaneously, monthly pulse rituximab therapy (1 g/month) was started for two months, followed by pulse therapy every six months. On the three-month follow-up, laboratory results revealed non-progression of kidney function (serum creatinine 1.22 mg/dl and urea 98 mg/dl) and a significant decline of almost 66% in proteinuria, with 24-hour urine collection showing 3.8 g/24h. However, more remarkable results were pointed out 12 months after the continuation of the above therapeutic scheme, while the patient was receiving the standard antihypertensive therapy, revealing a further decrease in the patient’s proteinuria with 24-hour urine collection showing protein excretion at 1.9 g, representing an 85% decline. Throughout the patient’s observation, values of serum creatinine remained invariable, reflecting satisfactory correspondence without worsening of kidney function, apart from a temporary mild decline in kidney function in the second month of follow-up, attributable to hemodynamic effects of dapagliflozin (Figure 2).

## Discussion

FGN is a rare immune complex-mediated glomerulonephritis mainly distinguished by the formation of organized, randomly arranged microfibrils (10-30 nm in diameter) in the glomeruli, representing a quite unusual etiology of nephrotic syndrome [3,7]. These microfibrils stain strongly by IF for IgG (mainly IgG4 subclass), IgM, IgA, C3, C1q, κ-, and λ- chains [4]. On electron microscopy, fibrillary depositions are mainly observed in the mesangium and the GBM, yet subendothelial and subepithelial zone depositions have also been described [4,8]. FGN fibrils differentiate from those observed in amyloidosis as being larger in diameter (8-10 nm in amyloidosis) and staining negative for Congo red [3,4]. However, Alexander et al. described a series of 18 cases of congophilic FGN in which the deposits were Congo red-positive and highlighted the role of DNAJB9 in distinguishing congophilic FGN from amyloidosis. In this research, the proteomic signature of amyloid was not detected using mass spectrometry among cases of congophilic FGN, and congophilia was supposed to be the result of increased amounts of apolipoprotein E (APOE) in the deposits [9].

Under the spectrum of Congo red-negative amyloid-like glomerulopathies, two main entities are described: FGN and immunotactoid glomerulopathy (ITG) [10-12]. Morphologically, the larger microtubules of ITG, measuring over 30nm in diameter and organized in stacked or parallel arrays with hollow centers, were easily distinguished from their counterparts in FGN on electron microscopy [3,4,13]. For many years, the distinction of these two glomerulopathies remained of controversial clinical importance [10-12].

Nowadays, we know that, except for the differentiation in the ultrastructural appearance, these two renal diseases have also distinct immunologic, pathophysiologic, and clinical features [10-12]. Deposits of IgG and C3 are present in both of these two glomerulopathies; however, the depositions usually receive a polyclonal pattern in FGN and a monoclonal one in ITG. Furthermore, ITG has been more often correlated with advanced age and hematopoietic and lymphoproliferative diseases when compared to FGN [10-12]. As for the prognosis, FGN is supposed to have a more rapid progression to ESKD than ITG and therefore poorer prognosis [10-12].

At the time of initial presentation, clinical manifestations include proteinuria in all patients, half of whom have nephrotic range proteinuria (mean 24-hour protein excretion at 5.62 g) and approximately one-third with apparent nephrotic syndrome features [3,4,8]. Kidney function impairment is noted in almost 70% of cases, with serum values of creatinine ranging from 0.5 to 8.3 mg/dl (mean values >2 mg/dl), while microscopic hematuria is present in the majority of patients (approximately 80%) [3,4,8]. Additionally, hypertension is clinically present in 50% of patients and peripheral edema is overt in almost 60% of all cases [4,8]. As described above, our patient presented as a hypertensive emergency without evidence of hypertension-mediated end-organ damage, apart from mild renal function decline in the setting of longstanding hypertension with poor compliance to antihypertensive therapy. Furthermore, the patient reported mild proteinuria that arbitrarily had been attributed to hypertensive nephrosclerosis and hyperfiltration due to former morbid obesity.

FGN is mainly a primary glomerular disease; however, approximately one-third of cases are associated with several other diseases. More specifically, diabetes mellitus seems to be the predominant comorbidity, noted in almost 20% of diagnosed FGN patients, while hepatitis C infection and dysproteinemia are present in 13%, respectively. Concurrent autoimmune disease (such as Grave’s disease, systemic lupus erythematosus, Crohn’s disease, idiopathic thrombocytopenic purpura, primary biliary cirrhosis, Sjogren’s syndrome or ankylosing spondylitis) coexist in approximately 10% [3,8]. Malignancies, including hematologic cancers (such as multiple myeloma, lymphomas, and leukemias) and solid tumors, have also been described as secondary causes of FGN in 9% of cases and the time of their diagnosis ranges between 15 years before and 10 years after the occurrence of renal involvement [3,8]. Some familiar cases, following an inherited autosomal dominant pattern, are also described in recent bibliography [14]. In this context, none of the above secondary causes of FGN was identified in our patient through extended investigation, including a bone marrow biopsy.

Indeed, there is no clinical or laboratory finding that is representative of FGN and only a kidney biopsy can totally confirm the diagnosis. Histopathological features on light microscopy can display heterogeneous patterns of glomerulonephritis, such as mesangial, membranoproliferative (MPGN), endocapillary proliferative, and crescentic or membranous-like glomerulonephritis [3,4,8,15]. The most common pattern seems to be mesangial glomerulonephritis followed by MPGN, while the most unusual is the membranous-like pattern presented in almost 2% of all cases [8]. Light microscopy findings include thickening of the glomerular capillary wall, variable hypercellularity of the mesangium, focal sclerosis, and immune-associated expansion of the mesangial matrix [4,8]. Interstitial fibrosis, tubular atrophy, focal findings of tubular injury, and arteriosclerosis are further characteristic features [4,8]. Congo red-negative staining remains the gold-standard finding in FGN biopsy specimens [3,4,8]. As regards our patient, the findings above on light microscopy masquerading as membranous glomerulopathy stage III-IV, compatible with the long-term effect of immune-complexes entrenchment, were observed, while a small kidney sample had been obtained, and more than that there was extensive involvement of GBM by fibrils.

IF reveals positive staining for immunoglobulins both on the capillary wall and the mesangium of the glomeruli [4]. The main immunoglobulin found in almost all biopsy specimens is IgG (mean intensity +3, range 0 to +4), with the predominant subclass being IgG4 in 100% of FGN biopsies, followed by IgM (mean intensity +0.8) in approximately two-thirds in IgA (mean intensity +1) in one-third of all cases [4,8]. Additionally, depositions of C3 subclasses of complement are present in over 90% of biopsy specimens, while C1q subclasses are found in almost 60% [8]. Kappa and lambda light-chain IF staining in the majority of cases is positive for both κ- and λ- chains; however, there are some rare cases with single staining of one of the light chains (more often observed single lambda-chain deposition rather than kappa-chain) [8]. This finding usually coexists with the presence of IgG-lambda and occasionally with the presence of IgG-kappa monoclonal bands in serum protein electrophoresis or immunofixation electrophoresis (SPEP/IFE) [8]. The above inconsistency was also present in our patient’s case, in which IgG-lambda depositions were present in IF staining while serum and urine IFE revealed IgG-kappa monoclonal bands.

The distribution of electron microscopy in the diagnosis of FGN is undoubtable, with the characteristic findings of fibrillary formations described above. Thus, recent research by immunohistochemistry has created a new significant path in FGN diagnosis. The identification of DNAJ homolog subfamily B member 9 (DNAJB9), a co-chaperone of the heat-shock protein 70 family members, which was first related to FGN in 2017, now serves as a 98% sensitive and, according to recent studies, 100% specific FGN biomarker. It is found in all biopsy specimens and almost 80% of the suspected cases [16,17]. DNAJB9 is mainly an intracellular protein, normally found in the endoplasmic reticulum of all cellular types. The only disease with extracellular presence of DNAJB9 is FGN, where DNAJB9 formates homogenous deposits in the mesangium and the capillary wall of the glomeruli [16-18]. The exact role of DNAJB9 in FGN pathogenesis has not been fully determined yet. However, the coexistence of DNAJB9 with IgG and complement depositions in the glomeruli advocates for autoimmune participation.

Data about the preferable therapeutic options in FGN are limited. Various therapeutic regimens have been used containing steroids as a monotherapy or combined with another immunosuppressive agent such as cyclophosphamide, cyclosporin, mycophenolate mofetil, azathioprine, lenalidomide, and rituximab, with the results being of doubtable benefit [3,8,19].

Additionally, many studies reviewed the outcomes observed after kidney transplantation in patients with FGN-associated ESKD. According to these studies, a high incidence of biopsy-proven disease recurrence in kidney allografts was reported in almost 50% of cases [20]. However, the evidence of recurrence is mostly characterized as benign with follow-ups showing satisfactory and non-progressive kidney function as prolonged kidney allograft survival [20].

As an autoimmune-mediated glomerulonephritis, FGN pathogenesis is associated with the activation of B cells. Therefore, the use of rituximab is supposed to offer a promising outcome. Recent research has suggested that rituximab therapy alone or with simultaneous steroid intake offers a non-progression in kidney function in a 12-month observation and a satisfactory reduction in 24-hour proteinuria [19]. However, no case of complete remission after rituximab therapy was recorded [17].

The simultaneous use of antiproteinuric and renal-protective therapy in kidney disease constitutes a common practice in daily clinical life. Angiotensin-converting enzyme (ACE) inhibitors and angiotensin II receptor blockers (ARBs) are the most well-tested agents used in kidney diseases, aiming to regulate intraglomerular hypertension and reduce proteinuria. SGLT2 inhibitors created a new path in the treatment of diabetes, while its beneficial role has also been extended both in heart and kidney failure [21]. Indeed, new studies brought to light that SGLT2 inhibitors have also given results in lowering nephrotic-range proteinuria in non-diabetic patients, helping the non-progression decline in estimated GFR, delaying the development of ESKD, and leading to improvement of kidney function while reducing renal inflammation and interstitial fibrosis [21].

In this case, we report the first patient with FGN, who was treated with a combination of steroids, rituximab, and dapagliflozin as a complementary nephroprotective and antiproteinuric agent while he was receiving the standard antihypertensive therapy including ARBs. The observed outcomes in our patient’s follow-up showed preservation of kidney function and a significant decrease, almost 85%, in proteinuria after 12 months of treatment. Taken together, the findings of this case study suggest the beneficial effect of immunosuppressive therapy in combination with an SGLT2 inhibitor and pave the way to another possible area of future research given that the optimal therapeutic strategy, leading to total disease remission, has not been identified yet.

## Conclusions

FGN is a rare immune-mediated glomerular disease characterized histologically by diffuse mesangial matrix expansion and thickening of glomerular basement membranes due to deposits. These deposits have fibrillary structures by electron microscopy and are negative for Congo red stains but positive for polyclonal immune deposits in IF. Therefore, FGN on light microscopic evaluation can receive various histological patterns and may follow a membranous-like pattern, especially when there is extensive involvement of GBM by fibrils. Currently, the identification by immunohistochemistry of a novel biomarker, DNAJB9, has created a new path in FGN diagnosis, especially when ultramicroscopic examination is not performed routinely but is a prerequisite for diagnosis of glomerular deposition disease.
